# Supplementary material for: Combined Transcriptome and Proteome Analysis Provides Insights into Petaloidy in Pomegranate
Source: Plants (Basel). 2023 Jun 21;12(13):2402. doi: 10.3390/plants12132402 (PMC10346766; doi:10.3390/plants12132402)
Supplement: Supplementary file 1 [file plants-12-02402-s001.zip › plants-2406471-supplementary2.pdf]

Supplementary Materials

Table S2. Primer sequences of qRT-PCR.

Gene name	Forward primer（5'-3'）	Reverse primer（5'-3'）
PgActin	AGTCCTCTTCCAGCCATCTC	CACTGAGCACAATGTTTCCA
CDL15_Pgr002932	CCAAGATAGCGGAAGCTGAC	GGAAGGAAGTTGCGGTCATA
CDL15_Pgr005610	GGATCGAGGACAAGAACAGC	CGGGAGGAGAAGATGATGAG
CDL15_Pgr007875	ACATAGGGGTGTGGCAGAAG	GACCAGTGGTTGATGTGTCG
CDL15_Pgr008188	CGGCAAGCTCTTTGAGTTCT	AGCCCTATCGGTGAGATCCT
CDL15_Pgr008799	GATCGACAGCATAACGAGCA	CTCCATCCAAACGTCCATCT
CDL15_Pgr020463	AGAACTCGAGGCCAGCTACA	CAAGCTGCTCCAGTTCCTTC
CDL15_Pgr021374	AGACGAGGCCTTGATCTGTG	AAGCCTTCACCCATCCTTCT
CDL15_Pgr026983	GGAGATGGAACTGCACAACA	GAGGCTGCCCAATATCGTAA
CDL15_Pgr028367	CAGACAGTGCGAACACGACT	CGAGCTGCTTCAGTTCCTTC

Tabal S3. Petaloidy related DAPs of ornamental pomegranate

Function category	Gene annotation	Gene ID
polysaccharide metabolism	xyloglucan endotransglucosylase/hydrolase（XTH）	OWM82065.1/CDL15_Pgr001639、OWM76301.1/CDL15_Pgr009947
	endoglucanase 6（EG6）	OWM72163.1/CDL15_Pgr018046、OWM85232.1/CDL15_Pgr028019
	pectate lyase（PEL）	OWM91545.1/CDL15_Pgr024869、OWM65463.1/CDL15_Pgr009053
Jasmonic acid signal transduction	Jasmonic acid-amido synthetas（JAR1）	OWM81738.1/CDL15_Pgr007776
	2-oxoglutarate-dependent dioxygenase（2-ODD）	OWM68674.1/CDL15_Pgr023639

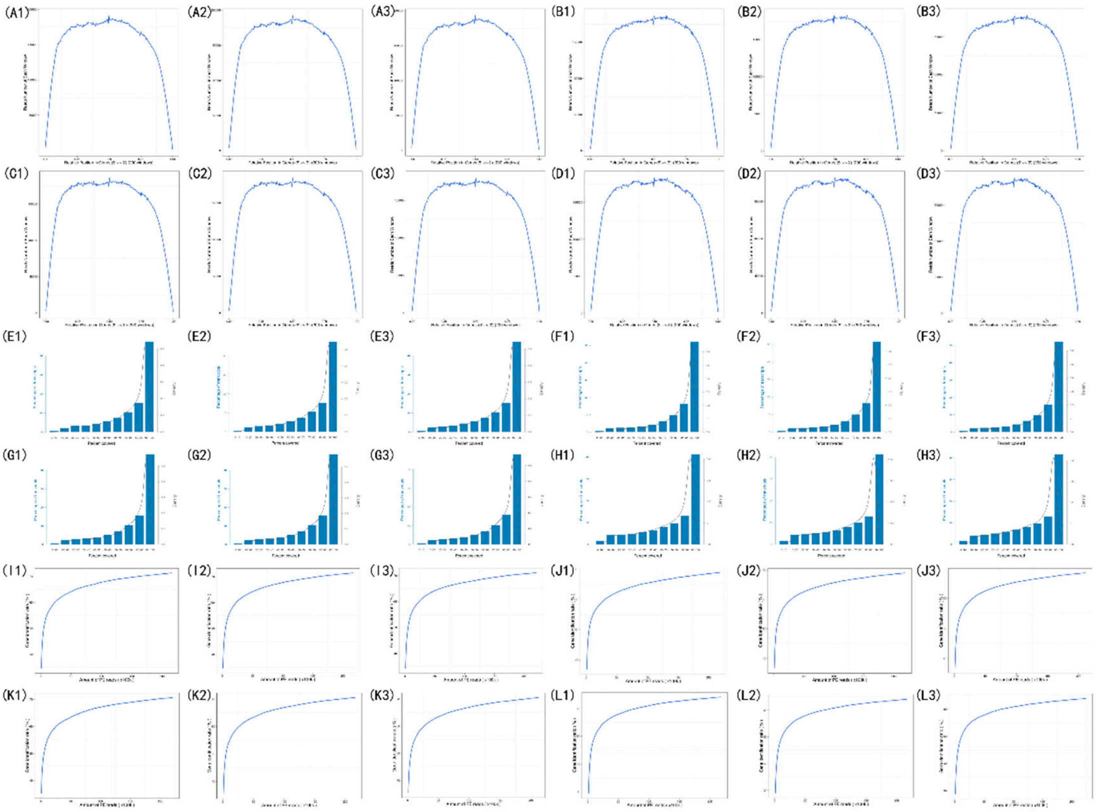

Figure S1. Quality control of mass spectrometry in ornamental pomegranate transcriptome. (a1–a3, b1–b3, c1–c3, and d1–d3) Random distribution map in StSi1, StSi2, StSi3, PeSi1, PeSi2, PeSi3, StDo1, StDo2, StDo3, PeDo1, PeDo2, PeDo3, respectively; (e1–e3, f1–f3, g1–g3, and h1–h3) Coverage distribution map in StSi1, StSi2, StSi3, PeSi1, PeSi2, PeSi3, StDo1, StDo2, StDo3, PeDo1, PeDo2, PeDo3, respectively; (i1–i3, j1–j3, k1–k3, and l1–l3) Sequencing saturation distribution map in StSi1, StSi2, StSi3, PeSi1, PeSi2, PeSi3, StDo1, StDo2, StDo3, PeDo1, PeDo2, PeDo3, respectively.

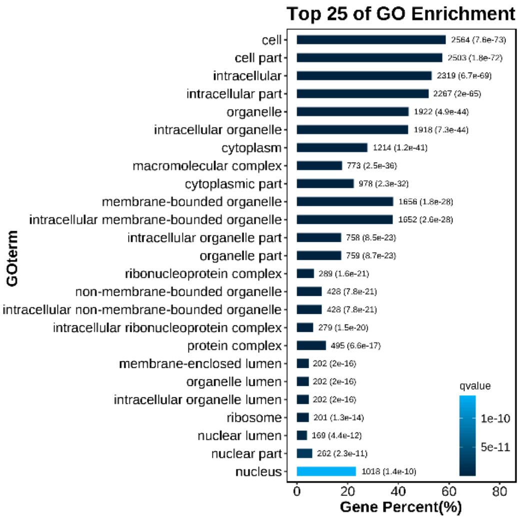

Figure S2. Enrichment map of GO cell components of DEGs in PeSi vs PeDo.

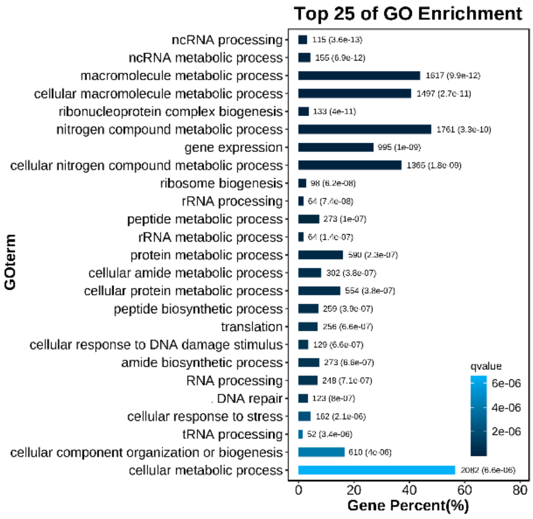

Figure S3. Enrichment map of GO biological process of DEGs in PeSi vs PeDo.

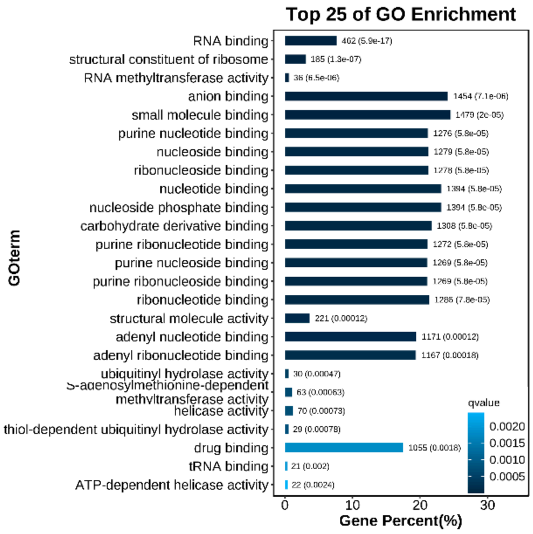

Figure S4. Enrichment map of GO molecular function of DEGs in PeSi vs PeDo.

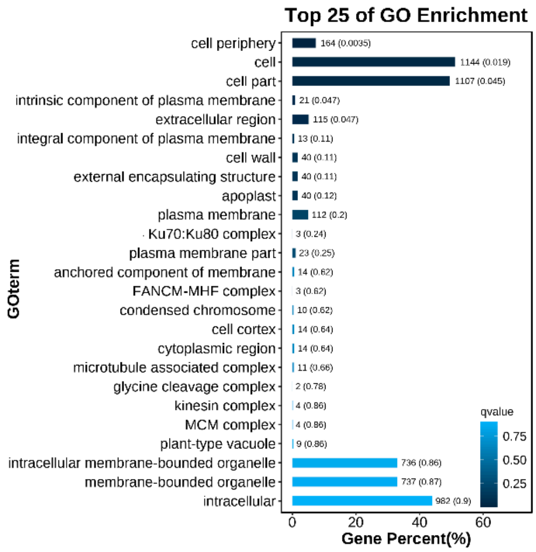

Figure S5. Enrichment map of GO cell components of DEGs in StSi vs StDo.

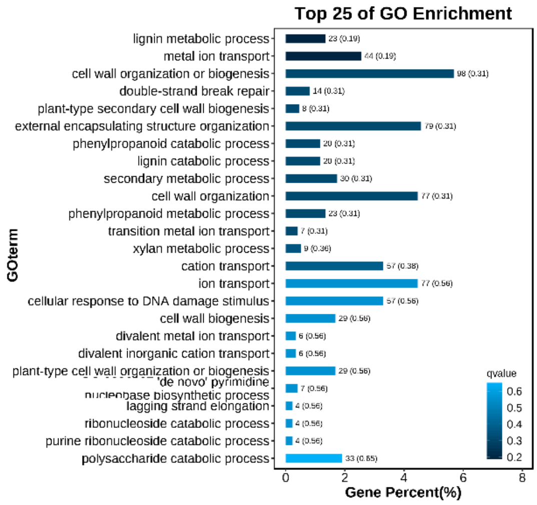

Figure S6. Enrichment map of GO biological process of DEGs in StSi vs StDo.

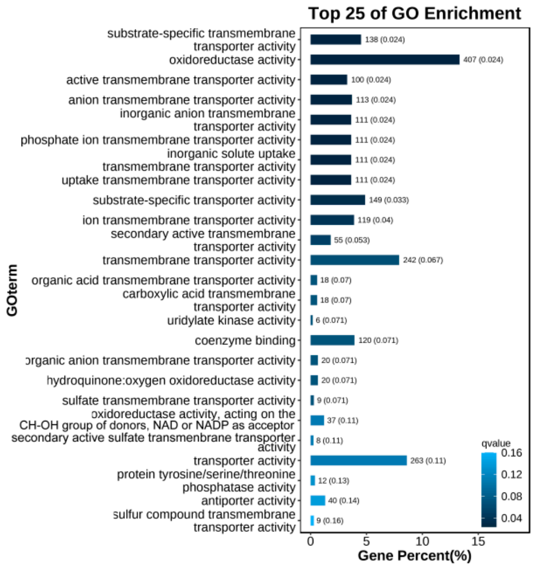

Figure S7. Enrichment map of GO molecular function of DEGs in StSi vs StDo.

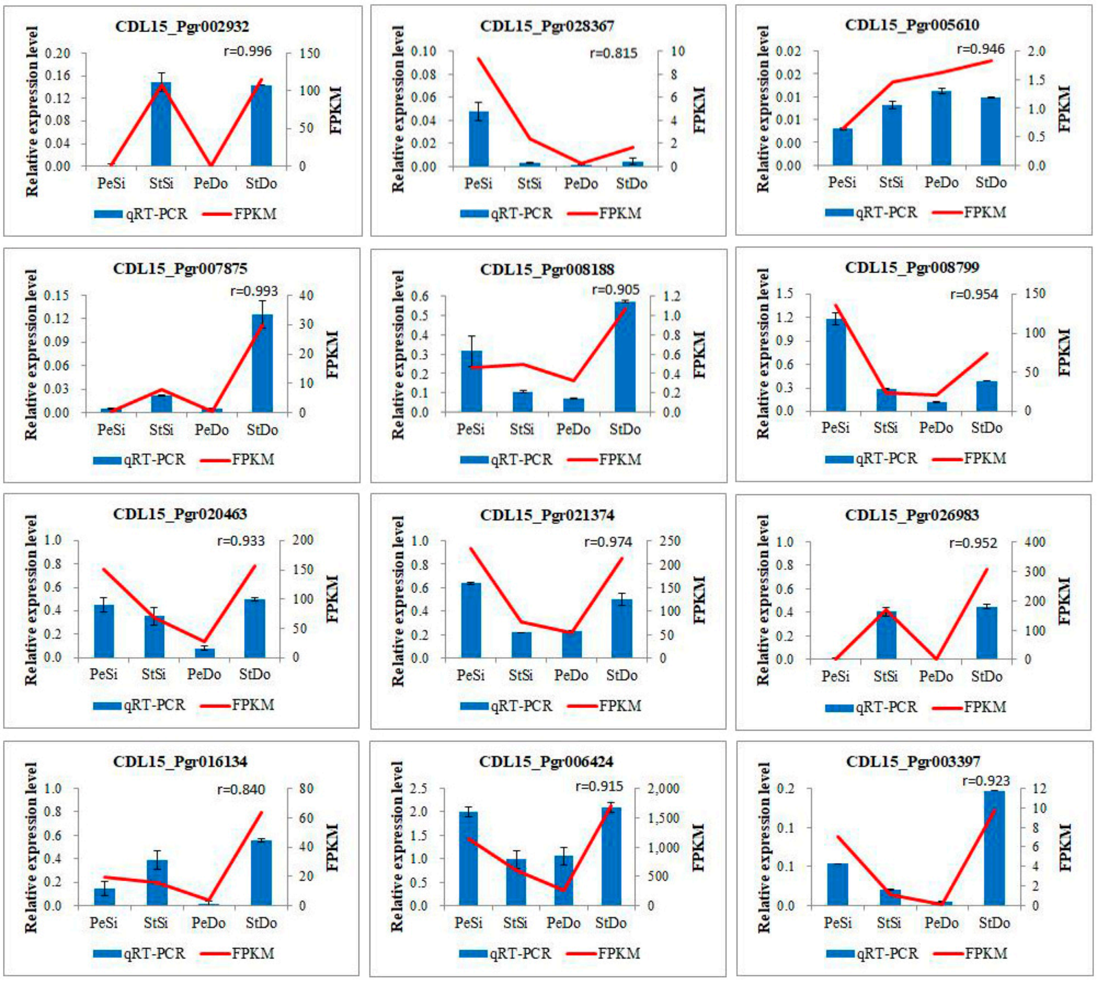

Figure S8. Validation of RNA-seq data using qRT-PCR.

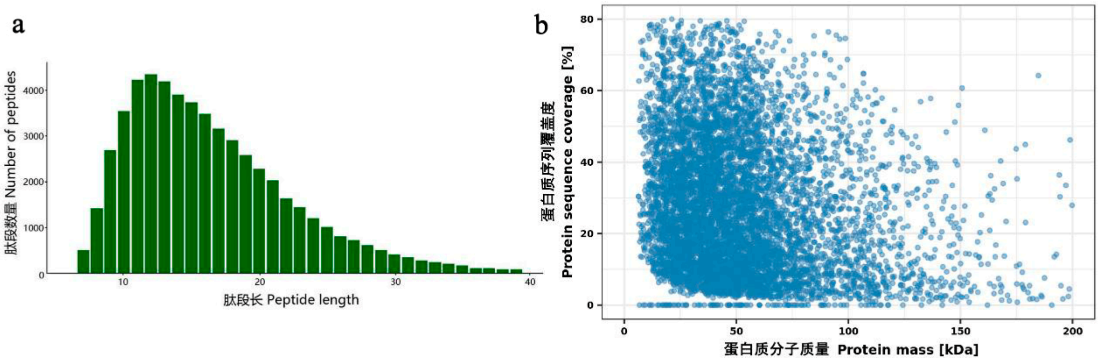

Figure S9. Quality control of mass spectrometry in ornamental pomegranate Proteome. Note: (a) Length distribution of identified peptides; (b) Molecular weight distribution of identified proteins.

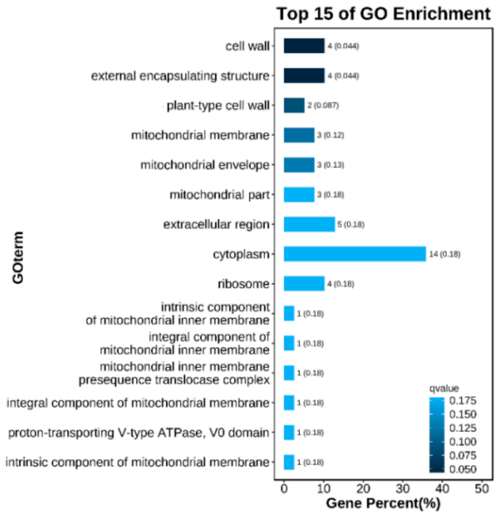

Figure S10. Enrichment map of GO cell components of petaloidy related DEPs.

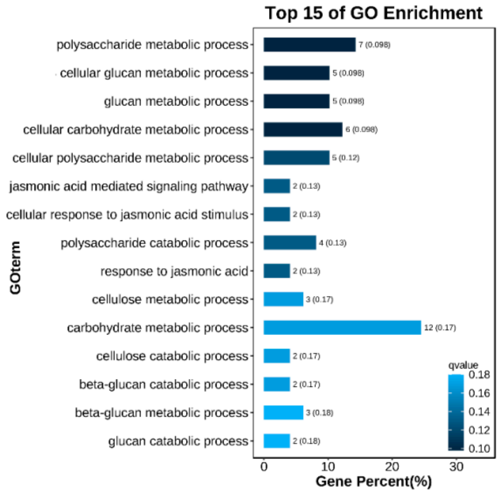

Figure S11. Enrichment map of GO biological process of petaloidy related DEPs.

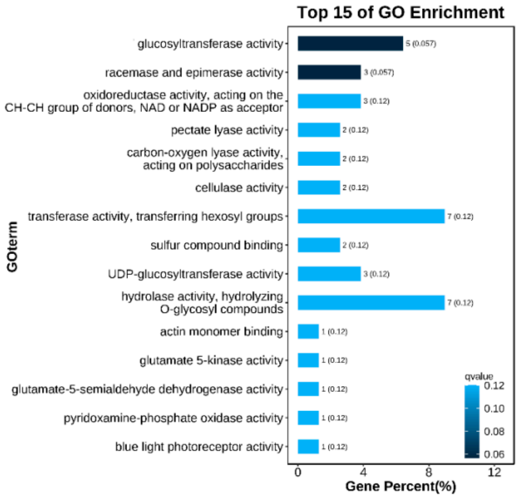

Figure S12. Enrichment map of GO molecular function of petaloidy related DEPs.

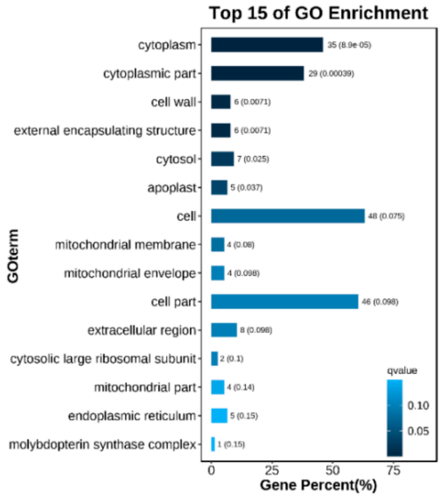

Figure S13. Enrichment map of GO cell components of DEGs /DAPs with the same expression trend

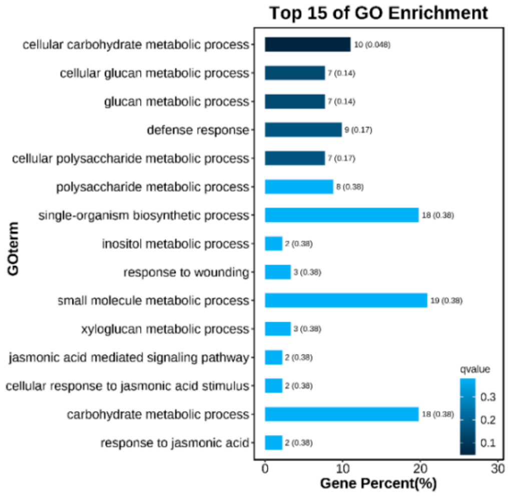

Figure S14. Enrichment map of GO biological process of DEGs /DAPs with the same expression trend

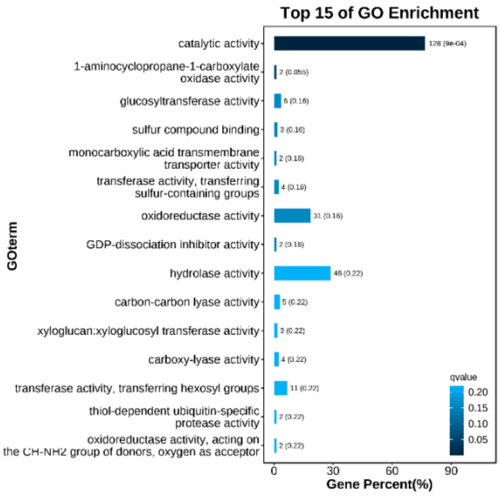

Figure S15. Enrichment map of GO molecular function of DEGs /DAPs with the same expression trend
